# Multiplex transcriptional analysis of paraffin-embedded liver needle biopsy from patients with liver fibrosis

**DOI:** 10.1186/1755-1536-5-21

**Published:** 2012-12-27

**Authors:** Nicholas R Staten, Eric A Welsh, Kurex Sidik, Sandra A McDonald, Dawn R Dufield, Botoul Maqsodi, Yunqing Ma, Gary K McMaster, Rodney W Mathews, Robert H Arch, Jaime L Masferrer, Bernard E Souberbielle

**Affiliations:** 1 Pfizer Global Research & Development, 700 Chesterfield Parkway West, Chesterfield, MO, 63017, USA; 2 Affymetrix, 3380 Central Expressway, Santa Clara, CA, 95051, USA; 3 Pfizer Worldwide Research & Development, Asia Research 575 Maryville Centre Drive, Saint Louis, MO, 63141, USA; 4 Pfizer Clinical Research, Ramsgate Road, Sandwich, Kent, CT139NJ, UK; 5 Present address: Kypha, Inc., 4320 Forest Park Avenue, St Louis, MO, 63108, USA; 6 Present address: GSK, Experimental Medicine Unit, Gunnels Wood Road, Stevenage, Herst, SG1 2NY, UK

**Keywords:** Liver fibrosis, Liver inflammation, Multiplex gene expression

## Abstract

**Background:**

The possibility of extracting RNA and measuring RNA expression from paraffin sections can allow extensive investigations on stored paraffin samples obtained from diseased livers and could help with studies of the natural history of liver fibrosis and inflammation, and in particular, correlate basic mechanisms to clinical outcomes.

**Results:**

To address this issue, a pilot study of multiplex gene expression using branched-chain DNA technology was conducted to directly measure mRNA expression in formalin-fixed paraffin-embedded needle biopsy samples of human liver. Twenty-five genes were selected for evaluation based on evidence obtained from human fibrotic liver, a rat BDL model and *in vitro* cultures of immortalized human hepatic stellate cells. The expression levels of these 25 genes were then correlated with liver fibrosis and inflammation activity scores. Statistical analysis revealed that three genes (*COL3A1*, *KRT18*, and *TUBB*) could separate fibrotic from non-fibrotic samples and that the expression of ten genes (*ANXA2*, *TIMP1*, *CTGF*, *COL4A1*, *KRT18*, *COL1A1*, *COL3A1*, *ACTA2*, *TGFB1*, *LOXL2*) were positively correlated with the level of liver inflammation activity.

**Conclusion:**

This is the first report describing this multiplex technique for liver fibrosis and has provided the proof of concept of the suitability of RNA extracted from paraffin sections for investigating the modulation of a panel of proinflammatory and profibrogenic genes. This pilot study suggests that this technique will allow extensive investigations on paraffin samples from diseased livers and possibly from any other tissue. Using identical or other genes, this multiplex expression technique could be applied to samples obtained from extensive patient cohorts with stored paraffin samples in order to correlate gene expression with valuable clinically relevant information. This method could be used to provide a better understanding of the mechanisms of liver fibrosis and inflammation, its progression, and help development of new therapeutic approaches for this indication.

## Background

Conducting gene expression analysis at the level of the organ of interest and correlating specific expression patterns to physiopathology, prognosis or early response to therapy remains a topic under intense investigation in liver fibrosis [1]. The ability to analyze gene expression from formalin-fixed paraffin-embedded (FFPE) tissue would be logistically useful because of access to banked samples which are usually paraffin-embedded specimens. For example, the description of this approach for hepatocellular carcinoma on paraffin-fixed tissue [2] has underscored the importance of the technology.

Here, we describe a multiplex gene expression pilot study using the bDNA technology QuantiGene™ Reagent System (Affymetrix, Santa Clara, CA, USA) for quantifying mRNA expression in FFPE liver samples [3]. Using this single multiplex assay, the expression levels of 25 genes were correlated with the extent of disease activity in fibrogenic chronic liver disorders. Included in the panel were genes known to be associated with liver fibrosis and hepatic stellate cell (HSC) activation, which have both been shown to correlate with inflammation [4]. In addition, several putative HSC housekeeping genes were chosen to assess for a correlation of HSC activity to stage of fibrosis or inflammation. Proof of concept was established showing that RNA extracted from paraffin sections could be used to investigate the modulation of a panel of proinflammatory and profibrogenic genes with this multiplex assay.

## Results

### Identification of gene expression panel

Previously performed bioinformatics analyses of whole genome microarray expression data identified 25 genes associated with fibrosis, HSC activation, or HSC housekeeping (Table 1). These expression data consisted of: 1) human fibrotic liver, 2) rat liver from a bile duct ligation fibrosis model, and 3) two separate human HSC activation models (LI90 and LX-2). The microarray data [5] are available from the ArrayExpress data repository (http://www.ebi.ac.uk/arrayexpress): human fibrotic liver (E-MEXP-2589), rat fibrosis BDL model day-7 (E-MEXP-2583), plastic-activated LI90 cells (Accession No. E-MEXP-2582), and plastic-activated LX-2 cells (E-MEXP-2584). Microarray data were analyzed through the use of IPA (Ingenuity® Systems, http://www.ingenuity.com). Genes were selected based on their upregulation or downregulation in fibrotic human and/or rat samples versus matched non-fibrotic samples as well as their upregulation in plastic activated LI90 and LX-2 HSC lines [6]. Putative HSC housekeeping genes were chosen if they: 1) were highly expressed in HSCs, 2) did not change significantly with HSC activation, and 3) had been described as specifically expressed in HSCs or poorly expressed in other hepatic cell types. Ultimately, we identified 19 targets associated with fibrosis and/or HSC activation and seven HSC housekeeping genes that appeared to be highly and specifically expressed in resting or activated HSCs. One gene, *TUBB*, was identified as both a marker of fibrosis from a rat fibrosis BDL model and as an HSC housekeeping gene from LI90 and LX-2 activated HSC models, thus, bringing the number of genes to 25. An additional five nonspecific assay housekeeping genes (*ACTB*, *TBP*, *RPL13A*, *PPIA*, and *UBC*) were recommended by the manufacturer of the QuantiGene™ Multiplex bDNA assay (Affymetrix, Santa Clara, CA, USA) to normalize expression of the 25-gene panel and therefore create a 30-plex gene expression array.

**Table 1:** A **30-plex gene expression panel, including five human gene normalization controls and the two human ribosomal (DNA and RNA) controls used in singleplex**

**Gene**	**Gene name**	**GenBank accession**	**Rationale**
18S ribosomal DNA	.	NR_003286	Tissue Quality & Quantity Assessment
28S ribosomal RNA	.	NR_003287	“ “
*ACTB*	Actin, beta	NM_001101	Normalization
*PPIA*	Peptidylprolyl isomerase A (cyclophilin A)	NM_021130	“ “
*RPL13A*	Ribosomal protein L13a	NM_012423	“ “
*TBP*	TATA box binding protein	NM_003194	“ “
*UBC*	Ubiquitin C	NM_021009	“ “
*ACTA2*	Actin, alpha 2, smooth muscle, *aorta*	NM_001613	Fibrosis and/or HSC Activation Marker
*ANXA2*	Annexin A2	NM_001002857	“ “
*ARNT2*	Aryl-hydrocarbon receptor nuclear translocator 2	NM_014862	HSC Housekeeping
*COL1A1*	Collagen, type I, alpha 1	NM_000088	Fibrosis and/or HSC Activation Marker
*COL3A1*	Collagen, type III, alpha 1	NM_000090	“ “
*COL4A1*	Collagen, type IV, alpha 1	NM_001845	“ “
*CPEB1*	Cytoplasmic polyadenylation element binding protein 1	NM_030594	HSC Housekeeping
*CTGF*	Connective tissue growth factor	NM_001901	Fibrosis and/or HSC Activation Marker
*CTHRC1*	Collagen triple helix repeat containing 1	NM_138455	“ “
*CXCL2*	Chemokine (C-X-C motif) ligand 2 ; CINC-2a, GROb, Gro2, MIP-2, MIP-2a	NM_002089	“ “
*EDN1*	Endothelin 1	NM_001955	“ “
*FOXF1*	Forkhead box F1	NM_001451	HSC Housekeeping
*HAMP*	Hepcidin antimicrobial peptide	NM_021175	Fibrosis and/or HSC Activation Marker
*HSPA1A*	Heat shock 70 kDa protein 1A	NM_005345	“ “
*IGFBP6*	Insulin-like growth factor binding protein 6	NM_002178	HSC Housekeeping
*KRT18*	Keratin 18; cytokeratin 18, cell proliferation inducing protein 46	NM_000224	Fibrosis and/or HSC Activation Marker
*LOXL2*	Lysyl oxidase-like 2	NM_002318	“ “
*MRGPRF*	MAS-related GPR, member F	NM_145015	HSC Housekeeping
*P311*	Neuronal protein 3.1, C5orf13	NM_004772	“ “
*SYP*	Synaptophysin	NM_003179	Fibrosis and/or HSC Activation Marker
*TGFB1*	Transforming growth factor, beta 1	NM_000660	“ “
*TGFB2*	Transforming growth factor, beta 2	NM_003238	“ “
*TIMP1*	TIMP metallopeptidase inhibitor 1	NM_003254	“ “
*TNF*	Tumor necrosis factor (TNF superfamily, member 2)	NM_000594	“ “
*TUBB*	Tubulin, beta	NM_178014	HSC Housekeeping & Fibrosis Marker

### HSC, hepatic stellate cells

#### Performance of the multiplex assay with FFPE liver samples

The technique was first standardized on cadaver liver tissue blocks. Five sections (60 mm^2^) of 8-μm thick sections from cadaver liver tissue blocks resulted in the most reproducible results. The 30-plex assay was then used to assess gene expression in all 25 needle biopsies. The optimum quantity of liver tissue for best sensitivity and reproducibility was equivalent to two to three needle biopsy sections (approximately 20 to 30 mm^2^) 8-μm thick for detection of all 30 genes tested. QuantiGene™ 2.0 multiplex intraassay coefficient of variations (CVs) were less than 10% for duplicate sections and less than 15% for triplicate sections. The interassay CVs were less than 20% for technical replicates. Data for four genes from the original set of 25 were discarded due to weak signal intensities across the majority of samples (*CXCL2* and *SYP*) or in more than 50% of samples (*ARNT2* and *CPEB1*). In addition, one of the QuantiGene™ assay normalization genes, *UBC*, was not included for further analysis due to inconsistent behavior across multiple strip testing.

Six genes were identified with significantly upregulated expression in fibrotic liver: *LOXL2*, *TUBB*, *COL1A1*, *TBP* (all *P* <0.0001), *COL3A1* (*P* = 0.0002), and *P311* (*P* = 0.0008). One gene showed significantly downregulated expression in fibrotic liver: *KRT18* (*P* = 0.0002). Among these seven differentially expressed genes, *TBP* was originally selected as a gene for assay normalization.

#### Development of a logistic model for assessing liver fibrosis progression

A logistic model was also established to determine those informative genes that could provide insight into liver fibrosis progression [7]. The disease status as a binary response was modeled on the covariates, that is, the gene expression measurements. First, each gene was evaluated separately by fitting the simple logistic model. From this preliminary one-gene-at-a-time modeling step, nine out of 25 genes were selected, including the seven genes listed above along with two additional genes: *ACTB* and *PPIA*. A logistical model was then built by utilizing the forward selection method based on the nine genes from the previous step using the SAS PROC LOGISTIC procedure [8]. Comparison of the modeling results with the *t*-test, principle component analysis (PCA), and partial least square (PLS) analyses resulted in the selection of three genes (*COL3A1*, *KRT18*, and *TUBB*,) as potential informative covariates. However, this logistical model needs to be tested with additional data in order to validate this model and establish it as a useful prediction model for liver fibrosis status.

#### Correlation between gene expression and disease activity

**Figure 1:**
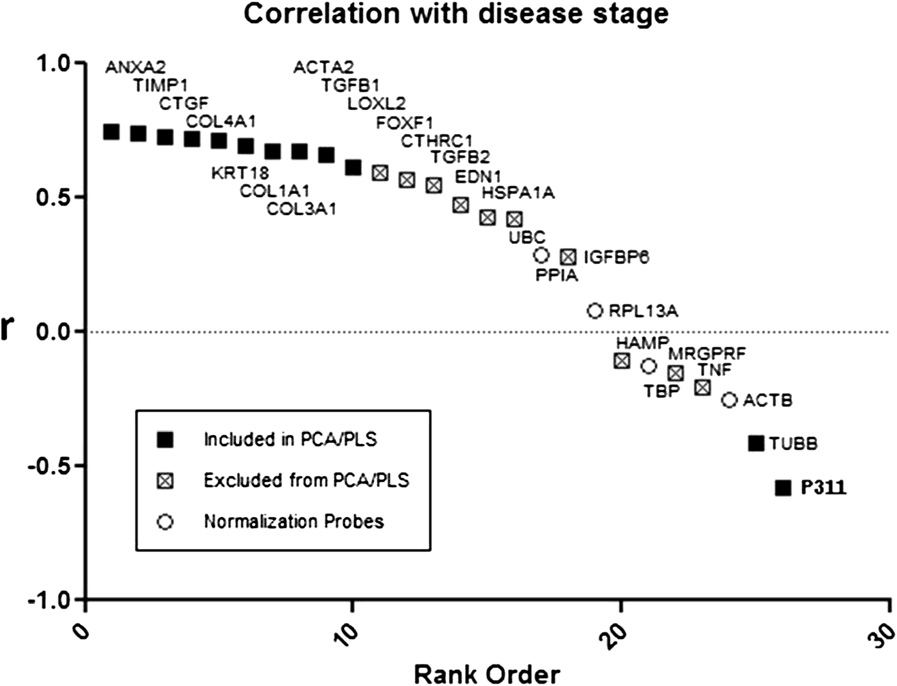
**Correlation with necroinflammation activity scoring.** The Pearson correlation (r) of the log10 (gene expression) vector with inflammation activity score is plotted versus the correlation rank order (computed correlation coefficient between the ranks of gene expression and inflammation activity) for each probe. The ten most highly correlated (positive) genes (_▀_) were selected for inclusion in the principle component analysis (PCA)/ partial least squares (PLS) models, as well as two anticorrelated (negative) genes (*TUBB*, *P311*) (_▀_).

**Figure 2:**
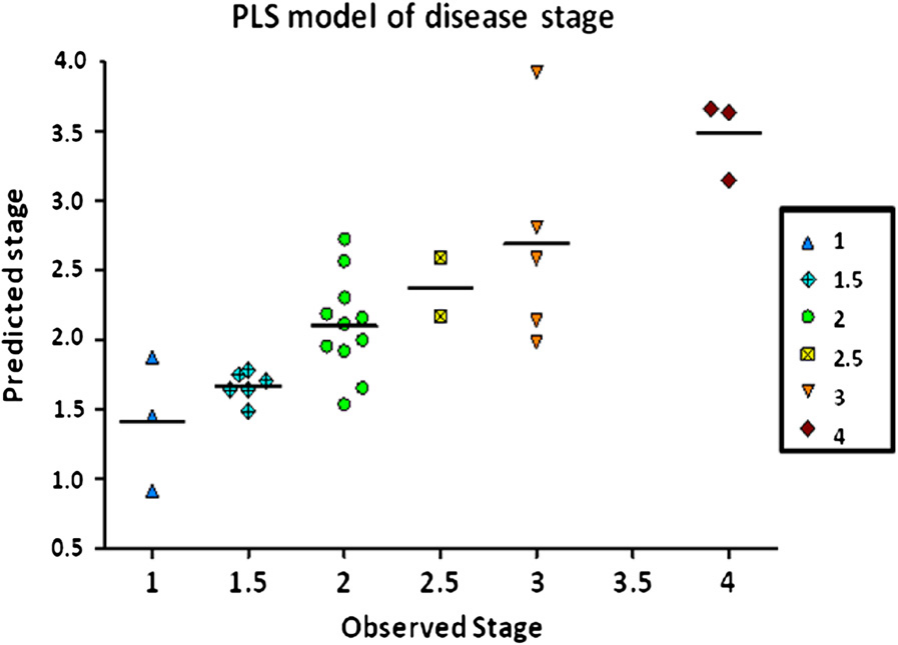
**Partial least squares model of necroinflammation activity grading.** A one-component partial least squares (PLS) model was trained on the twelve selected genes (ten positive and two negative correlated) versus observed necroinflammation activity stage within the diseased needle biopsy samples alone (R^2^: 0.69, Q^2^: 0.66). Necroinflammation stage is predicted to within approximately ±0.5 stage.

Regardless of the number of components, no correlation with the stage of fibrosis (Q^2^, the cross-validated R^2^) could be detected. However, the expression of 12 out of 21 genes (57%) showed either strong positive (*ANXA2*, *TIMP1*, *CTGF*, *COL4A1*, *KRT18*, *COL1A1*, *COL3A1*, *ACTA2*, *TGFB1*, *LOXL2*) or negative (*TUBB*, *P311*) correlation with necroinflammation score (Figure 1). These 12 genes were selected as yielding the most discriminant PCA and PLS models. The two negatively correlated genes chosen as HSC housekeeping genes, *TUBB* and *P311*, did not perform as well as the other 10 genes in the PLS analysis, but contributed to the separation of controls from disease samples in the PCA analysis. The two-component PCA captured 76% of the variance, with a cross-validated (leave 1/7^th^ out) Q^2^ of 0.63 (data not shown). Given the correlation with disease activity observed within diseased samples, a single-component PLS model was built within the diseased samples using the selected 12 probes. The resulting model was able to predict the activity grading of tissue samples of fibrogenic chronic liver diseases with a R^2^ of 0.69 and Q^2^ of 0.66 with a maximum error of approximately ±0.5 stage (Figure 2). Inclusion of a second component in the PLS model was not statistically justified.

## Discussion

This report describes a pilot study evaluating a multiplex gene analysis assay, relevant for complex diseases with limited tissue availability, which proved reliable for the study of tissue samples of fibrogenic chronic liver diseases using FFPE needle biopsy samples. The optimum quantity of liver tissue for best sensitivity and reproducibility was equivalent to two to three needle biopsy sections 8-μm thick for detection of all genes tested. Using this approach, the expression of ten genes, known to be associated with liver fibrosis and/or HSCs activation, were identified as positively correlating with necroinflammatory activity, whereas two HSC housekeeping genes were identified as negatively correlating with activity, though we could not find a strong correlation between gene expression and fibrosis stage.

We used the bDNA technology QuantiGene™ Reagent System for quantifying mRNA expression. The technical advantage of the sandwich nucleic acid hybridization assay [3] lies in amplification of reporter signals without enzymatic amplification of the target mRNA, which allows direct measurement of mRNA in tissue homogenates, overcoming issues associated with mRNA extraction. This technology was chosen over a qPCR-based assay method for two primary reasons: 1) QuantiGene™ detection can accurately measure mRNA in FFPE samples whereas qPCR cannot reliably measure mRNA, which has an average size of 100 bases; and 2) QuantiGene™ can be multiplexed more easily than qPCR which is more suited for expression analysis of mRNA extracted from fresh or frozen samples. Prior attempts to employ qPCR on a subset of selected genes in paraffin resulted in no detectable gene expression in paraffin liver RNA and resulted in no detectable gene expression or gene expression beyond the limit of detection (Staten, data not published). Thus, we were not able to use qPCR to compare gene expression between samples or correlate between qPCR and bDNA technologies.

The selection of the genes to be evaluated was made arbitrarily on the basis of a mixed combination of data obtained from human fibrotic liver, a rat BDL model and *in vitro* culture of immortalized hepatic stellate cells, namely L190 and LX2, and therefore the selected genes are likely related to established inflammation and fibrosis. Other genes could be used in this multiplex assay to address complementary, but different, questions. For example we did not select all of the candidate genes strictly on the basis of a subtraction analysis done by comparing gene expression in fibrotic liver (or activated HSC) and normal liver (or quiescent stellate cells), as described by others previously [9,10]. This alternative approach may be more appropriate to study the difference of gene expression between disease state and normal liver. However, our approach and the choice of genes used in the present study are more appropriate for investigating the modulation of inflammation and fibrosis between different samples of diseased liver, and could also be applied to verify the modulation of these genes following a period of treatment (for example, predictor of antifibrotic and anti-inflammatory response).

Prognostic factors of rapid progression of liver fibrosis to cirrhosis based on liver biopsy, but also on somatic genetic markers, have been described [11,12]. For example, Asselah and colleagues [13] identified 11 genes that differentiate mild from moderate fibrosis based on their respective expression levels. But only two of these genes (*COL1A1*, *TIMP1*) overlapped with our study. For identification of potential therapeutic targets, this multiplex assay can be customized with other genes of interest. Liver fibrosis is a disease characterized by collagen deposition [14] and we included in the multiplex assay multiple genes coding for proteins involved in extra cellular matrix formation, deposition and turnover (*COL1A1*, *COL3A1*, *COL4A1*, *ACTA2* and *TIMP1*); and one gene encoding a component of the cytoskeleton (*KRT18*). However, in our report, the highest correlation with inflammatory activity stage was observed for annexin 2 (*ANXA2*). *ANXA2* has many described functions but its role in the hepato-biliary system is not fully understood. It is expressed in many cells including hepatocytes, HSCs [15] and cholangiocytes [16]. *ANXA2* is involved in the plasminogen/plasmin system with fibrinolytic effects promoting clot dissolution in alcoholic liver cirrhosis [15]. In inflammatory cholangiopathy *ANXA2* can mediate a compensatory effect for the impaired bicarbonate secretion of cholangiocytes through modulation of anion exchanger [16]. *ANXA2* has been shown to be expressed as a vitamin D binding protein on hepatocytes and HSCs. Some of these interactions could be important for liver fibrogenesis and inflammation through a TGFβ-independent mechanism [17].

## Conclusions

In conclusion, the results of this pilot study provide feasibility evidence (proof of concept) for the use of multiplex gene expression methods on FFPE samples from chronic fibrotic liver tissues. However, because of possible limitations of our study (choice of genes, limited samples used for analysis, scoring and grading done only with METAVIR), these results are not yet conclusive for the definition of the diagnostic potential of the panel of genes employed. Indeed, we were unable to source FFPE fine needle biopsies from nonfibrotic control livers. Furthermore, our analysis includes two distinct tissue sources, that is, cadaveric samples (fibrotic and nonfibrotic livers) and needle biopsies from fibrotic livers. Thus, the time to process samples from cadavers between time of death and time of processing (4 to 10 hours post mortem) may impact the mRNA levels of specific hepatic genes. Therefore, our results should be corroborated by further studies including an adequate number of cases thoroughly characterized in terms of etiology, grading and staging, and with adequate sampling conditions. Following this demonstration, the same technical multiplex scheme could be applied to samples obtained from extensive cohorts with stored paraffin samples to obtain more precise and clinically relevant information. In particular, it will be possible to correlate gene expression (employing a customized multiplex gene panel), with histopathology (stage and activity), clinical and biochemical data, and prognostic indexes (time to cirrhosis, liver related events, survival, *etcetera*) in retrospective or prospective analysis.

## Methods

### Fixed human liver samples

Human FFPE block samples were acquired from various external academic sources in compliance with Pfizer’s human tissue policy and approval by the Institutional Review Board of the source institution. Samples were de-identified and patient identities were not traceable by Pfizer at any point in time. Needle biopsies from fibrotic livers were collected from 18 HCV and seven non-alcoholic steatohepatitis (NASH) patients (14 male and 11 females; 23 to 65 years of age). To the best of our knowledge, these biopsies were fixed within 0.5 hours of procurement and processed through surgical pathology at the home institutions. In addition, we obtained 11 nonfibrotic and five (HCV-negative) fibrotic liver samples collected from cadavers fixed with a lag-time of 4 to 10 hours postmortem. These FFPE blocks were sectioned (8 μm thick), stained with hematoxylin and eosin, and transferred into 1.5 mL microcentrifuge tubes for further processing [18].

### Fibrosis and activity scoring

All of the hematoxylin and eosin-stained liver samples were assessed by a single pathologist at Pfizer for inflammatory activity and changes in tissue architecture using scoring systems adapted from the Knodell system for inflammation [19] and the METAVIR scoring system for fibrosis [20]. Inflammation grading was indicated as: 0 = no inflammation, 1 = minimal inflammation, 2 = mild inflammation, 3 = moderate inflammation, and 4 = marked/severe inflammation, with half point scores used at the pathologist’s discretion. The presence of necrotic hepatocytes was accounted for in the overall grading scheme. Fibrosis grading was indicated as: 0 = normal architecture, 1 = minimally expanded portal tracts, 2 = periportal fibrosis, 3 = bridging fibrosis, and 4 = cirrhosis.

### QuantiGene™ multiplex bDNA assay for the 30-plex gene expression panel

FFPE tissue homogenates were prepared as previously described [21]. Briefly, FFPE sections were homogenized in tissue homogenizing solution (THS) (Affymetrix Inc. Santa Clara, CA) supplemented with 500 μg/mL Proteinase K and incubated for 6 hours at 65°C. The tissue homogenate was processed immediately or stored at −80°C until use.

For the cadaver liver tissue blocks, five sections of 60 mm^2^ (each 8 μm thick) resulted in the most reproducible results (data not shown). These sections were homogenized individually in 300 μL of THS and digested with 500 μg/mL Proteinase K for 6 hours at 65°C. For needle biopsy samples, two sections from each of the liver needle biopsies ranging from approximated 20 to 30 mm^2^ (8 μm thick) were homogenized in 150 μL THS using 500 μg/mL proteinase K for 6 hours at 65°C. The mRNA quality and quantity were assessed using the 18S rRNA probe set (1 μL homogenate) and 28S rRNA probe set (0.001 μL homogenate) in duplicate on all homogenates [21]. Finally, 40 μL of homogenate of each sample were tested in triplicate using the 30-plex gene panel.

The multiplex bDNA assays were run as recommended in the QuantiGene™ Plex 2.0 Reagent System User Manual. Probe design software [22] was used to generate probe sets for singleplex or multiplex bDNA assays [3]. Probe sets were designed and synthesized by Affymetrix, Inc. (Santa Clara, CA, USA). Samples containing 40 μL of homogenate were mixed with pooled multiplex probe sets and capture beads (2,000 beads per gene per assay) prior to hybridization in 100 μL total volume, for 20 hours at 55°C. Hybridization reactions were transferred to a 0.45 μm filter plate (Millipore, Billerica, MA, USA), followed by sequential hybridization at 55°C with the bDNA amplifier and 5^′^-dT(biotin)-conjugated label probe. Unbound materials were washed from beads complexed with probe set and mRNA by alternating filtration and the addition of wash buffer (0.1X SSC, 0.03% lithium lauryl sulfate). Two washes were performed after each step. After the final wash, streptavidin-conjugated R-phycoerythrin (SAPE) was added and incubated at room temperature for 30 minutes. Analysis was performed after an additional wash to remove unbound SAPE.

### Data analysis

Background signals (relative light units) were determined in the absence of RNA and subtracted from signals obtained with RNA, setting any negative signals to zero. Background subtracted signals were used to calculate the coefficient of variation (CV) and sensitivity of all assays, as well as for normalization of the RNA expression of each gene. Sensitivity was evaluated by determining the limit of detection, defined as target RNA concentration at which the signal is three standard deviations above background. Data were normalized by four assay housekeeping genes (*TBP*, *ACTB*, *PPIA*, and *RPL13A*) using the geometric mean [23] for each sample. The *UBC* assay housekeeping gene was omitted due to inconsistent behavior across multiple strip assays.

### Statistical analysis

In order to identify significantly differentially expressed liver fibrotic genes, a screening approach was first followed to compare fibrotic (sourced from needle biopsy and cadavers) and nonfibrotic liver tissue (sourced only from cadavers) using the two-sample *t*-test on the gene expression data of 26 genes (excluding four genes of the 30 genes for analysis: *SYP*, *CXCL2*, *ARNT2*, and *CBEP1*). Considering multiplicity testing issues, comparisons were controlled using the false discovery rate at the 5% test level [24]. In order to correlate liver fibrosis and inflammation stages with gene expression profiles, principle component analysis (PCA) and partial least-squares analysis (PLS) (Evince software package, Umbio, Umeå, Sweden) were performed on log transformed intensities, scaled and centered to unit variance.

## Abbreviations

CV: coefficient of variation; ECM: extra cellular matrix; FFPE: formalin-fixed paraffin-embedded; HCV: hepatitis C virus; HSC: hepatic stellate cells; NASH: non-alcoholic steatohepatitis; H&E: hematoxylin and eosin; PCA: principle component analysis; PLS: partial least square; qPCR: quantitative PCR; RNA: ribonucleic acid; THS: tissue homogenizing solution; SAPE: streptavidin-conjugated R-phycoerythrin; THS: tissue homogenizing solution.

## Competing interest

BM, YM and GKM are employees of Affymetrix, which has developed the multiplex technique described in this paper.

## Authors’ contributions

NS, EW, KS, SM, DD, BM, GM, RM, MJ contributed to the design, acquisition of data, analysis and interpretation and to the draft of the manuscript. NS, RA and BS contributed also to the analysis and interpretation of the data, and directed and drafted the manuscript. All authors read and approved the final manuscript.

## Authors’ information

RA’s current address: GlaxoSmithKline (China) R&D Co., Ltd. Building 3, 898 Halei Road. Zhangjiang Hi-Tech Park, Pudony. Shangai 201203, China.

DD’s current address: Pfizer Global Research & Development, 1 Burtt Rd, Andover, MA 01810, USA.
